# Interactions of Constipation, Dysfunctional Elimination Syndrome, and Vesicoureteral Reflux

**DOI:** 10.1155/2008/828275

**Published:** 2008-06-04

**Authors:** Sarel Halachmi, Walid A. Farhat

**Affiliations:** ^1^Department of Urology, Rambam Medical Center, The Faculty of Medicine, Technion-Israel Institute of Technology, Haifa 31096, Israel; ^2^Division of Urology, Hospital for Sick Children, Faculty of Medicine, University of Toronto, Ontario, Canada M5G 1X8

## Abstract

Vesicoureteral reflux (VUR) is simply described as incompetence of the unidirectional valve at the ureterovesical junction (UVJ), leading to backflow of urine to the kidney. Today, it is clear that VUR is not only related to the UVJ function but also to a combination of processes including immunity, bladder and pelvic floor function, dysfunctional voiding, and constipation. Although our surgical aims directed towards improving the valve coaptation at the UVJ, we understand today the importance of the diagnosis and treatment of constipation and dysfunctional voiding adjunctively.

## 1. INTRODUCTION

The frequency of stools in most children decreases from a mean of four per day in the first week of life to 1.7 per day by the age of 2. Over this interval, stool volume increases more than tenfold, while maintaining a consistent water content of approximately 75%, and the intestinal transit time from mouth to rectum increases from 8 hours in the first month of life to 16 hours by the age of 2 to 26 hours by the age of 10 [1, 2].

Constipation generally is defined by the hard nature of the stool, the pain associated with its passage or the failure to pass three stools per week [1]. In most children, functional fecal retention is the most common chronic disorder of defecation caused by the voluntary withholding of feces due to fear of painful defecation. Many events may lead to unpleasant defecation such as aggressive toilet training; for instance, as children approach toilet-training age, the child's gratifying impulse to defecate is in conflict with parental constraints to establish bowel control and a premature or excessive parental emphasis on continence may lead the child to the decision to withhold the stools [3]. Other causes for fecal holding are changes in routine or diet, an intercurrent illness, or postponing the defecation because the child is too busy or toilets are unavailable [3, 4]. Once the child has experienced the painful passage of hard stool, he or she attempts to avoid the expected discomfort by exerting voluntary withholding. The rectum accommodates to the contents and the urge to defecate gradually vanishes. As the cycle is repeated, greater amounts of hard stool are built up in the rectum and pass with even greater pain, frightening the child [5, 6].

It has been reported that 34% of toddlers in the UK and 37% of Brazilian children younger than 12 years were considered by their parents to be constipated [7]. In the absence of painful symptoms, parents are generally unaware of the bowel habits of children older than 4 or 5 years. It is our experience that parents paid little attention to the frequency of their child's bowel movements, but paid much attention to the number of urinary and fecal incontinence episodes.

Encopresis or fecal soiling is usually the result of looser stool leaking or overflowing from a rectum that has been distended by retained stool; soiling occurs whenever the child tries to expel gas or the muscles which are used to withhold become fatigued. While encopresis is three to six times more common among males than females [1], we observed that voiding position may contribute to this higher prevalence of soiling in boys, as they stand to void, thus soiling in their underwear unknowingly. We speculate that the difference in females is that they remove their underwear and will sit on the toilet to void. Thus, the natural voiding position has a direct impact on the statistics of encopresis in boys compared to girls.

## 2. IMPACT OF CONSTIPATION ON THE URINARY SYSTEM

There are many reports correlating constipation with functional bladder-outlet dis-coordination being responsible for urinary incontinence, urinary tract infections, vesicoureteral reflux, and even false uroradiologic pathology [8–11]. The impacted stool in the rectum compresses the bladder, reduces its functional capacity, and provokes earlier sensation to void. In addition, chronic pelvic floor spasm prevents complete relaxation during voiding, and will attribute to postvoid residuals. Klijn et al. [12] showed that the diameter of the rectum in patients with dysfunctional voiding is higher compared to asymptomatic healthy children. The average rectal diameter in the dysfunctional voiding and constipated patients was 4.9 cm versus 2.1 in the control group (*P* < .001)

Kasirga et al. [13] compared 38 children concerning chronic functional constipation to 31 healthy children and found significant higher frequency of urinary tract infection and urgency. Neumann et al. [14] found that 34% of children with urinary tract infections had abnormal bowel patterns, and most parents were not forthcoming with this information. O'Regan et al. [11, 15] noted that constipation was associated with recurrent urinary tract infections and bladder instability in girls. They found that 50% of the mothers denied constipation to be present in their children, but questioning of the individual children revealed that most had only 2 to 3 bowel movements per week. O'Regan et al. [11, 15] also showed that children with enuresis had constipation. In 22 children with enuresis, rectal examination and rectal manometric studies proved constipation. Treatment of constipation resulted in resolution of enuresis. Uninhibited bladder contractions, observed in enuretic constipated children, were also noted in children with constipation alone, suggesting that constipation is a commonly unrecognized etiologic factor in enuresis [16].

Loening-Baucke [7] evaluated the frequency of urinary incontinence and urinary tract infection in 234 chronically constipated and encopretic children before, and after the start of treatment for constipation. Followup, at least 12 months after commencement of treatment, revealed that the constipation was relieved successfully in 52% of the patients. Relief of constipation resulted in disappearance of daytime urinary incontinence in 89% of the patients, nighttime urinary incontinence in 63% of the patients, and disappearance of recurrent urinary tract infections in all patients who had no anatomic urinary abnormalities.

## 3. CONSTIPATION DYSFUNCTIONAL ELIMINATION SYNDROME AND REFLUX

Numerous studies connected between vesicoureteral reflux and dysfunctional elimination syndrome (DES). O'regan et al. [17] correlated the directly established association of reflux, uninhibited bladder conduction, and constipation. The author examined urodynamic and rectal manometry on 17 children with VUR. All 17 had rectal dilatation and uninhibited bladder contraction confirming the vicious connection of these 3 conditions.

Koff et al. [10] assessed the influence of functional bladder and bowel disorder on the natural history of children with primary reflux. The authors assessed 143 children who had either spontaneous resolution of VUR or (82%) had breakthrough infection that led to definitive surgical management. Among patients without DES, only 18% had breakthrough infections and surgical correction. The rate of DES was higher in children with breakthrough infections, 77% versus 23%. The spontaneous resolution of reflux was longer in an average of 1.6 years in children with DES. Moreover, unsuccessful surgery or development of de novo contralateral reflux and post-successful surgery urinary tract infections appeared only in patients with DES. Care should be taken in the interpretations of the results of this paper as patients were selected to this study based on the presence of reflux. Naseer and steinhardt [18] demonstrated deleterious effect of dysfunctional voiding on VUR treatment end point—the development of scars. Among 538 with DES, 192 (13.5%) already had scars at initial assessment, 31 (2.1%) developed new scars despite meticulous care by the urology clinic. Eleven of those were referred to surgical treatment, however 6 developed new scars despite successful surgery.

Silva et al. [19] tried to identify independent factors predicting the resolution of VUR. DES was one of the 4 independent predictors for VUR resolution. The author concluded that treatment of DES is a cornerstone in the management of VUR. Upadhyay et al. [20] too demonstrated the relationship between DES, DES management, and the resolution of VUR. Using a DES standardized symptom score which included questions regarding bladder and bowel habits, the authors showed direct correlation between improvement in the symptoms score and resolution of VUR. In 7 patients with spontaneous resolution of VUR, there was also statistically significant symptom score improvement, in addition 4 patients showing the same symptom score improvement had also reduction of at least 2 grades in VUR grading. Improvement monitored by symptom score showed compliance to behavioral therapy and could predict VUR improvement and resolution.

In contrast to the above-mentioned studies, Sheikh et al. [21] assessed the relationship between early UTI, DES, and VUR. The authors examined 248 children, 123 of them had culture-proven UTI diagnosed by the age of 2. Control group consisted of 125 patients to whom urinalysis was performed due to fever ruled out UTI. Questionnaire given to all children showed no significant presence of DES (22% versus 21%). In patients with UTI who were later diagnosed with VUR, DES was present in 18% compared to 25% of those without VUR. The authors concluded that neither UTI nor VUR diagnosed before the age of 2 was associated with DES in school-aged children. The rate of DES is lower in patients with UTI and VUR compared to those without VUR, however there is still a significant percentage of patients with UTI and VUR who had DES. Chen et al. [22] studied 2759 patients who had renal sonography and voiding cystogram for various clinical entities. The authors used multivariate logistic regression approach to quantify the associations between DES and other pediatric urology factors. They showed that DES is present in 36%-36.1% of girls with unilateral and bilateral VUR, and in 20.5–21.2% of boys, respectively. The higher rate of DES in girls was independent of UTI and VUR status. In patients that diagnosed with VUR due to other entities beside UTI (sibling VUR, prenatal hydronephrosis, etc.), the association to DES was negative. Although this evidence may oppose the hypothesis that relationship between DES and VUR exists, however we think that these results clearly reflects the variety of patients categorized under VUR. Indeed, sibling VUR and VUR detected for other reasons rather than UTI is usually considered less significant, and the lower rate of related DES and UTI may give the explanation why. In this study, patients with VUR and UTI had almost doubled risk of DES supporting our belief that DES plays a major role in the pathophysiology of VUR.

## 4. CONCLUSIONS

Constipation plays a major role in the function and dysfunction of the urinary tract. Patients with constipation have concomitant urinary tract problems including infection, incontinence, enuresis, and VUR. There are strict evidences that constipation is related to the presence of reflux, urinary tract infections, breakthrough infections, presence of scars, and the appearance of new scars. Patients with constipation and DES have lower and longer rates of spontaneous resolution of reflux and vice versa, adherent to behavioral treatment, and improvement of the constipation is related to spontaneous VUR resolution.

Evaluation and treatment of constipation and DES should be an integral part of the initial assessment and management of a child with VUR, it would be also advocated to postpone definite surgical correction in patients with severe DES in order to improve surgical outcome.

## References

[1] Abi-Hanna A, Lake AM (1998). Constipation and encopresis in childhood. *Pediatrics in Review*.

[2] Lu C-L, Chen C-Y, Chang F-Y, Lee S-D (1998). Characteristics of small bowel motility in patients with irritable bowel syndrome and normal humans: an Oriental study. *Clinical Science*.

[3] Issenman RM, Filmer RB, Gorski PA (1999). A review of bowel and bladder control development in children: how gastrointestinal and urologic conditions relate to problems in toilet training. *Pediatrics*.

[4] Solzi G, Di Lorenzo C (1999). Are constipated children different from constipated adults?. *Digestive Diseases*.

[5] Loening-Baucke V (1993). Chronic constipation in children. *Gastroenterology*.

[6] Loening-Baucke V (1993). Constipation in early childhood: patient characteristics, treatment, and longterm follow up. *Gut*.

[7] Loening-Baucke V (1997). Urinary incontinence and urinary tract infection and their resolution with treatment of chronic constipation of childhood. *Pediatrics*.

[8] Farhat W, Bägli DJ, Capolicchio G (2000). The dysfunctional voiding scoring system: quantitative standardization of dysfunctional voiding symptoms in children. *Journal of Urology*.

[9] Koff SA (1992). Relationship between dysfunctional voiding and reflux. *Journal of Urology*.

[10] Koff SA, Wagner TT, Jayanthi VR (1998). The relationship among dysfunctional elimination syndromes, primary vesicoureteral reflux and urinary tract infections in children. *Journal of Urology*.

[11] O'Regan S, Yazbeck S (1985). Constipation: a cause of enuresis, urinary tract infection and vesico-ureteral reflux in children. *Medical Hypotheses*.

[12] Klijn AJ, Asselman M, Vijverberg MAW, Dik P, de Jong TPVM (2004). The diameter of the rectum on ultrasonography as a diagnostic tool for constipation in children with dysfunctional voiding. *Journal of Urology*.

[13] Kasirga E, Akil I, Yilmaz Ö, Polat M, Gözmen S, Egemen A (2006). Evaluation of voiding dysfunctions in children with chronic functional constipation. *The Turkish Journal of Pediatrics*.

[14] Neumann PZ, DeDomenico IJ, Nogrady MB (1973). Constipation and urinary tract infection. *Pediatrics*.

[15] O'Regan S, Yazbeck S, Schick E (1985). Constipation, bladder instability, urinary tract infection syndrome. *Clinical Nephrology*.

[16] O'Regan S, Yazbeck S, Hamberger B, Schick E (1986). Constipation a commonly unrecognized cause of enuresis. *American Journal of Diseases of Children*.

[17] O'Regan S, Schick E, Hamburger B, Yazbeck S (1986). Constipation associated with vesicoureteral reflux. *Urology*.

[18] Naseer SR, Steinhardt GF (1997). New renal scars in children with urinary tract infections, vesicoureteral reflux and voiding dysfunction: a prospective evaluation. *Journal of Urology*.

[19] Silva JMP, Diniz JSS, Lima EM, Vergara RM, Oliveira EA (2006). Predictive factors of resolution of primary vesico-ureteric reflux: a multivariate analysis. *BJU International*.

[20] Upadhyay J, Bolduc S, Bägli DJ, McLorie GA, Khoury AE, Farhat W (2003). Use of the dysfunctional voiding symptom score to predict resolution of vesicoureteral reflux in children with voiding dysfunction. *Journal of Urology*.

[21] Shaikh N, Hoberman A, Wise B (2003). Dysfunctional elimination syndrome: is it related to urinary tract infection or vesicoureteral reflux diagnosed early in life?. *Pediatrics*.

[22] Chen JJ, Mao W, Homayoon K, Steinhardt GF (2004). A multivariate analysis of dysfunctional elimination syndrome, and its relationships with gender, urinary tract infection and vesicoureteral reflux in children. *Journal of Urology*.

